# Birth experience from the perspective of the fathers

**DOI:** 10.1007/s00404-020-05714-z

**Published:** 2020-08-01

**Authors:** Lena C. Vischer, Xenia Heun, Joscha Steetskamp, Annette Hasenburg, Christine Skala

**Affiliations:** Department of Gynecology and Obstetrics, Mainz University Medical Center, Langenbeckstr. 1, 55131 Mainz, Germany

**Keywords:** Childbirth, Posttraumatic stress disorder, Obstetric violence, Paternal emotions during, Labor

## Abstract

**Purpose:**

As men nowadays often attend the delivery of their own child, they also have to go through the labor period. In this study, the condition of the expectant fathers attending labor was evaluated.

**Methods:**

In 2016, fathers who went through labor with their partners in the University medical center of Mainz were interviewed within the first days after delivery and 6 months later. They received a “Fathers questionnaire” concerning their motivation, the valuation of their attendance, their emotions during labor, and concerning the service in the labor ward. Six months later, they also received the validated Impact of Event Scale questionnaire (IES-R). In total, 318 participants answered the “Fathers’ Questionnaire”, 226 the IES-R.

**Results:**

Father’s attendance during labor was considered to be beneficial for fathers themselves (254, 79.8%), for the mother (272, 85.5%), for the newborn child (187, 58.8%), for the relationship (234, 73.6%). Only four could not see a purpose in their attendance. 73 men (23%) felt helpless, 47 (14.8%) were overwhelmed by the situation, 116 (36.5%) felt fear, 299 (94%) were happy to be present at birth, 27 (8.5%) felt traumatized by experiencing their partners in labor. According to the IES-R, none of the 226 men surveyed showed all symptoms of post-traumatic stress disorder.

**Conclusion:**

Childbirth is related to positive and negative emotions. Positive emotions are predominant, but come along with negative feelings. In this survey, posttraumatic stress disorder did not occur among men after delivery. Nevertheless, fathers’ needs should be kept in view.

## Introduction

Childbirth is ideally the embodiment of happiness felt by the parents of a newborn child. However, delivery is a border experience in the life of a woman. During childbirth, a woman is at the mercy of this special situation, due to the nature of childbirth, including the experience of pain and sometimes the first confrontation with hospitalisation and medical intervention. As men often participate in the birth of their own children, this event represents a fundamental turning point in the lives of both, women and men.

When a child is born, it will require both parents’ full attention. A major change takes place both in everyday life and on the emotional level. Transition to parenthood is ideally accompanied by many positive feelings. However, not every birth is identical and uncomplicated. Many births are different from parents’ own expectations and wishes. When complications arise, promptness is often required on the part of medical staff in the delivery room to ensure the health of mother and child. There is no time to adapt, and the situation can be overwhelming for both parents. In this context, there is a permanent discussion about obstetric violence [1, 2]. Traumatic experiences can be the result, which might affect the upcoming months or years [3]. This applies not only to women, but also to the respective partner [4].

First of all, after a highly traumatic situation, there is a temporary shock or an acute stress reaction. If coping strategies exist, the negative experience can be processed in the following weeks and the symptoms will lessen. Postpartum depression is far better researched in the literature than post-traumatic stress disorder related to childbirth. However, this is an important topic with a significant effect on the contact between both parents and the newborn as well as on the parents’ relationship. The prevalence of paternal postnatal depression is estimated to be at least 8.2–12% [5–8]. Literature figures out, that, when studies have been carried out focusing on birth and the effects on fathers, the focus lies primarily on post-traumatic stress disorder and less on birth experience itself. Focusing on post-traumatic stress disorder only highlights a negative, i.e., traumatic, aspect of childbirth. Positive feelings are neglected, although they are among the essential and desirable emotions. In particular, the emotional budget of the father-in-be during birth should be examined and their birth experience investigated. In our study, we solved this task using a newly created partner questionnaire.

The present study is focused on the experience of fathers during labor. Since there is still very little literature on the paternal, postpartum post-traumatic stress disorder, we also examined this issue using the validated Impact of Event Scale questionnaire [9]. Therefore, we tried to find out, why men decided to accompany their partners during labor and how they felt at the labor ward of University Medical Center Mainz, Germany.

## Methods

As a matter of quality management, we initiated the following verification of the public accusation of obstetric violence in delivery rooms. We performed a 1-year prospective longitudinal analysis from January to December 2016. Mothers and their partners were interviewed on the topic of “Birth experience” at two different points in time T1 and T2. T1 was within the first 5 days after birth. Data at time T2 were collected 6 months later.

The fathers received a collection of questions, which is called the “Fathers’ Questionnaire”, (Appendix) concerning their motivation (questions 1–4), concerning the benefit of their attendance (questions 5–8), their emotions during labor (questions 9–14), and concerning the service of the labor room of the University Medical Center in Mainz (questions 15, 16). Six months after birth, the fathers were also given the validated IES-R questionnaire.

In 2016, a total of 1989 births took place in the Department of Obstetrics and Women's Health at the University Medical Center in Mainz. At time T1, 382 partners received the questionnaire of which 185 returned. Six months later, the second survey took place and the questionnaires were sent to 590 fathers and 226 responses returned by mail. 93 partners answered the questionnaire at both times. In total, 318 men completed the “Fathers’ Questionnaire”, while 226 also completed the IES–R questionnaire.

PTSD score was met put into relation to obstetric parameters such as mode of delivery and duration of labor.

### Statistics

Statistical analysis was performed using Statistics Package for Social Sciences (SPSS 22.0, Chicago IL; USA).

Descriptive analysis took place using total numbers and percentages for categorial variables, while means with standard deviations were given for continuous variables. Besides, a correlation to obstetric parameters was performed with Spearman’s rank correlation coefficient.

All patients gave written consent for participating in this evaluation. It was performed in accordance with the ethical standards of the Johannes Gutenberg University Mainz and with the 1964 Helsinki Declaration and its later amendments or comparable ethical standards.

## Results

**Fig. 1:**
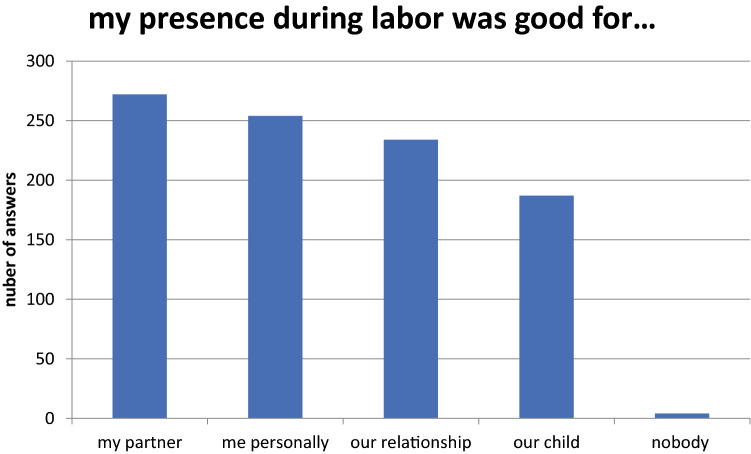
The answers of the fathers about their impression, for whom their presence during labor was good
  Questions concerning the feelings
  73 (23.0%) felt helpless, 245 (77.0%) did not. 47 men (14.8%) were overwhelmed by the situation, 271 (85.2%) were not. 116 (36.5%) felt fear, 202 (63.5%) did not. 299 men (94.0%) felt happy to be present at birth, 19 were not (6.0%). 27 men (8.5%) felt traumatized by labor, 291 (91.5%) did not. 306 men (96.2%) decided to participate at the birth of their next child. Twelve (3.8%) would prefer not to attend labor (Fig. 2).

1. *Fathers’ Questionnaire*
  211 out of 318 men (66.4%) witnessed the birth of their first child.
  Questions concerning the motivation
  There were different motivations of participating in labor. 287 fathers wanted to attend the birth of their own accord; in 196 cases, their presence was the wish of the prospective mother.
  63 out of 318 men (19.8%) had no idea what to expect when attending the childbirth [255 (80.2%) did]. In 75 fathers (29.4%) who had notions what to expect during labor, these expectations did not come true.
  Questions concerning the use of their attendance
  268 men (84.3%) felt involved in the birth, 50 (15.7%) did not. The fathers were asked if their attendance during labor was good. 272 (85.5%) answered that it was good for their partner, for 254 (79.8%), it was good for themselves, 187 (58.8%) had the impression that it was good for the newborn child. 234 (73.6%) participants expected their attendance to be beneficial for their relationship. Four fathers (1.25%) had the impression that their attendance at labor was not beneficial (Fig. 1). 15 men (4.7%) had the impression that their partner did not need them during labor, (303 did, 95.3%). 281 men (88.4%) thought they could support their partner well, 37 (11.6%) did not.

**Fig. 2:**
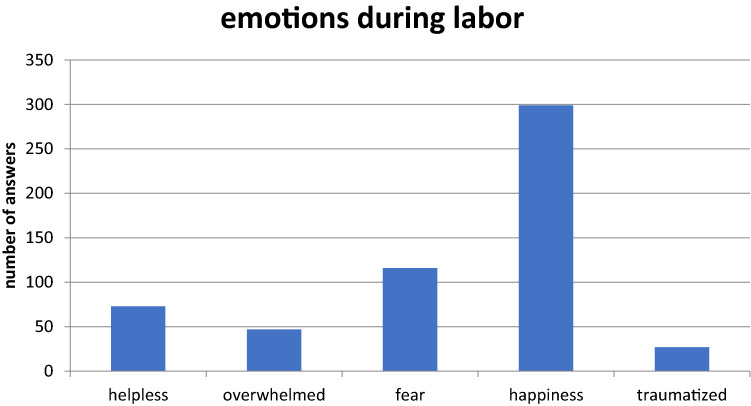
The fathers’ emotions during labor
  Questions concerning the service in the delivery room
  We asked two open questions. The fathers could give a comment, on what should be improved. There were many suggestions. Some men felt trouble seeing the partner suffering from labor pain. Other fathers would have liked to spend more time together with mother and child after birth. Some partners wished more interpersonal care on the part of the delivery room staff. Own needs were also addressed. Some wanted a more comfortable armchair or a supply of food and drink. Even the desire for hard liquor was expressed, which shows the extended tension.
2. *Impact of event scale*
  Six months after birth, the new fathers were evaluated with the Impact of Event Scale questionnaire. The IES-R consists of 22 questions, seven questions dealing with intrusion, eight questions with avoidance, and seven questions with hyperarousal. A score is calculated and lies in between a spread of − 4.36 and 2.99. A score > 0 gives evidence for a PTSD.
  None of the 226 men reached a score above zero. Therefore, none is deemed to suffer from PTSD. Intrusion, avoidance and overexcitation were regarded separately, to evaluate men’s individual stress level.
  Intrusion was most noticeable: 162 fathers (71.7%) had no signs of intrusion. 51 men (22.6%) showed a moderate form of intrusion and five men (2.2%) suffered from a severe form. Most of the men (207, 88.5%) showed no or at the most a slight avoidance. Moderate and severe avoidance was hardly observed, only in eight (3.5%) and two men (0.9%). Similar results were obtained concerning overexcitation. Most fathers had no or only a low score for overexcitation (202, 89.4%). A moderate form was found in 7.3% (14 men), severe overexcitation in 0.9% (two men).
  The PTSD score was put in relation to obstetric parameters (Table 1).

**Table 1:** PTSD score in relation to the mode of delivery and the duration of labor

	Spontaneous delivery	Cesarean section	Vacuum extraction	Total	Correlation to PTSD score
Mode of delivery	141	54	18	217	*p* = 0.25
PTSD score	− 4.10	− 3.78	− 3.89	− 4.0	
Duration of labor (min)	255.42	95.01	260.67	215.58	*p* = 0.53

Figure 3

**Fig. 3:**
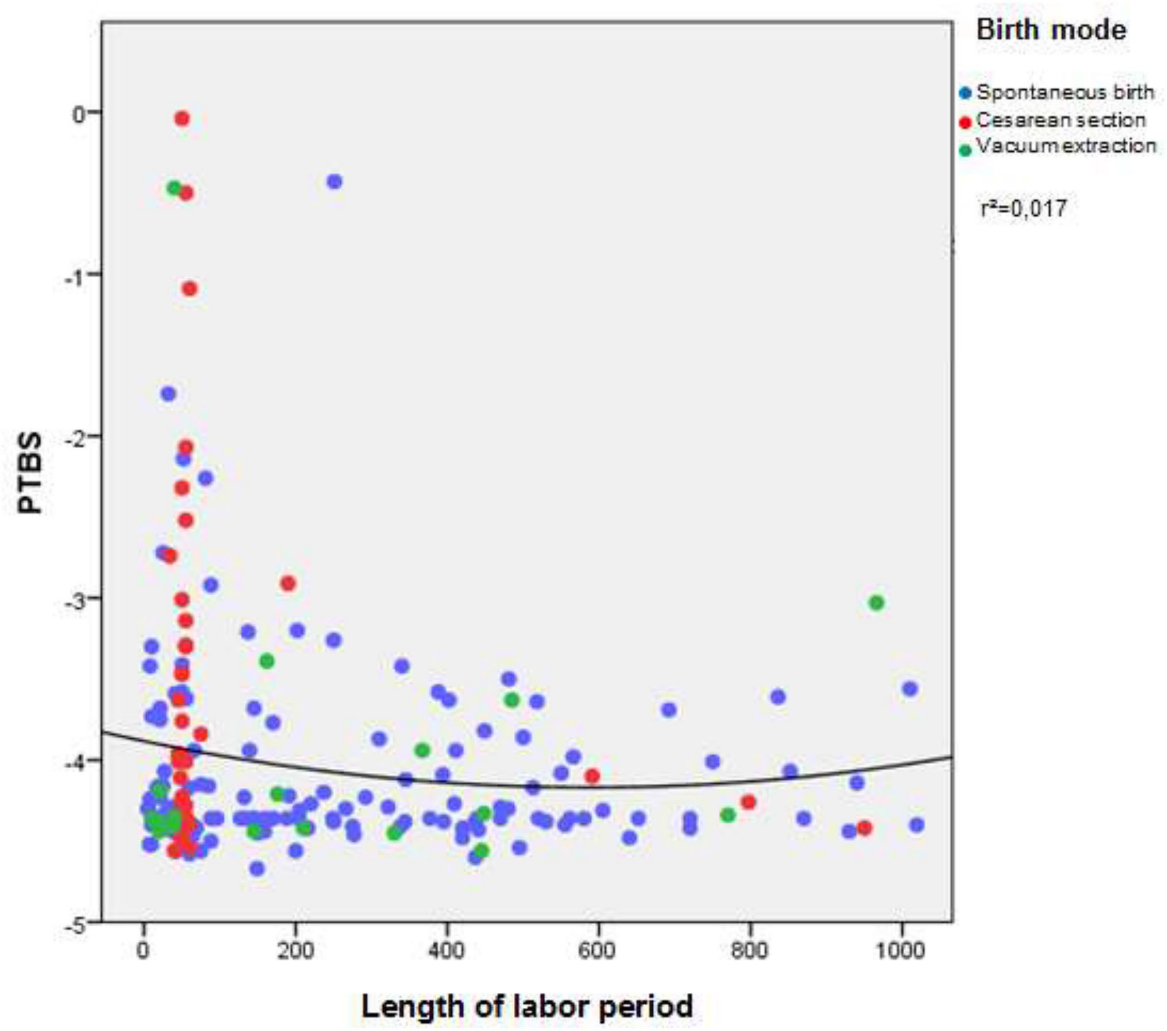
presents the PTSD score in relation to the length of labor period. The cut off for PTBS is > 0. Therefore, it is obvious that none of the probands showed PTBS. Concerning the length of the birth, very short labors seem to be more stressful

presents the PTSD score in relation to the length of labor period. By trend, short labors seem to be more stressful for attending partners. Anyhow, length of childbirth was not significantly correlated with the PTSD expression score (Spearman’s rho: − 0.038, *p* = 0.533). Furthermore, the PTSD expression score did not differ significantly whether the woman had given childbirth naturally, by means of primary, secondary or emergency cesarean section, or by vacuum extraction (Kruskal–Wallis-H: 5.417, *p* = 0.247). Eventually, the influence of an emergency cesarean section on paternal PTSD score levels could not be measured, since there was only one case of this obstetrical condition. Distribution of intrusion levels (0–8: mild, 9–19: moderate, 20 and above: heavy) did also not differ significantly between these groups (*X*^2^: 7.946, *p* = 0.439).

## Discussion

Overall, we found that most fathers had a positive birth experience and symptoms of intrusion, overexcitation and avoidance were only seen in a few men. Not a single father showed all symptoms of a PTSD. Our evaluation figures out that birth is an emotional event in fathers’ lives. Happiness was the predominant sentiment during labor. But negative feelings such as fear and helplessness are also present at birth either for the attending partner. These authentic negative emotions do not necessarily lead to a PTSD. The absolute majority would choose to be part of a birth again. However, 12 men (3.77%) prefer not to attend childbirth anymore. Most fathers advise fathers-to-be to be present at birth, because, in their opinion, there is hardly a comparable, more formative experience in a father’s life.

In total, most fathers experience positive feelings in retrospect concerning the birth of their own child. They were grateful and happy to have been present at birth and experienced a positive impact on the couple and on the relationship with the child. This coincides with the statements of Szeverenyi et al. [10] and Awad et al. [11]. There is a closer emotional connection between the couple after birth. However, the strongest feeling arises among men whose partners expressed the need for their emotional support in this situation. Most fathers felt, they were able to act as supportive and empowering partner during childbirth. The supportive part plays a prominent role in the emotional experience of the man [10, 11]. The feeling of fear has to be judged critically: many fathers experience a feeling of fear during birth, but most fathers feel neither helpless nor overwhelmed. Thus, the feeling of fear does not necessarily have to be linked to feelings such as helplessness or excessive demands during the birth. A recent Australian study [12] found that anxiety is more common than depression and is the most common diagnosis for men in the first 6 months after birth. In this trial, 17% of all men showed symptoms, while in 4.1% of all cases, an anxiety disorder was diagnosed [12]. Potapova et al. [13] argue that fatherly fear can deteriorate early childhood development.

First of all, this study shows that women’s pain is a crucial issue for fathers. It appears to be difficult for men to deal with the situation, seing their partner suffer and not being able to support them sufficiently. Two other studies came to similar conclusions, postulating the difficulty for men to stand helplessly next to the woman who is experiencing severe pain [14, 15]. In addition, quite often more support from medical staff is wished. Fathers-in-be seem to demand especially more opportunities to ask questions and to gather information. It would be good if the midwives were more responsive to the partner's needs and more involved in the birth process. The men signal that they would like to give their partner more active support. This is confirmed by Sapkota et al. [14]: the more the expectant fathers are integrated, the more they have the impression that they can adequately support their partner. However, since most partners are not very familiar with the process of giving birth, guidance by medical staff is necessary. Partners want more specific tasks. Bäckström et al. [16] came to similar results. This is also confirmed by the individual answers of the attending partners in this evaluation. Besides, more comfort in the labor rooms they wished good care and more presence of the medical staff.

None of the fathers showed all symptoms of PTBS. Looking at intrusion, avoidance and overexcitation separately intrusion is predominant 6 months after birth. The subgroups avoidance and overexcitation are too small to allow interpretations. An explanation of why intrusion is particularly pronounced compared to the other two subgroups could be that the birth is perceived with all the senses: intrusion is defined by the reliving of situations or images and noises that arise in indefinite situations [5]. The birth experiences are still very present 6 months after the birth through their memorable events and through the change that a birth brings in the life of the parents. The images of birth can therefore be replayed for a long time in the father’s mind and the changing situation is often relived. Based on the results, one can assume that afterwards birth is processed more intensively than it is suppressed.

Other studies came to different results: two studies showed the overexcitation subgroup to be most pronounced. Only a few fathers were affected by the intrusion and avoidance subgroups [17, 18]. It seems that experiencing a high level of agitation during childbirth is a prerequisite for a post-traumatic response.

Risk factors for the development of PTSD such as younger age, anxious personality, economic problems, unwanted pregnancy have been described several times in the literature [4, 17]. This study gives a hint that there might be other risk factors. A short labor period goes along with a higher level of PTSD scores. As cesarean sections take less time, this surgical birth seems to be more stressful.

## Conclusion

Childbirth is related to positive and negative emotions for both parents. In terms of the father, positive emotions are predominant. However, negative sentiments are present in every forth or third man. Fortunately, a posttraumatic stress disorder did not occur. Medical staff in the delivery room has a great impact on the wellbeing of fathers and mothers. Therefore, fathers’ needs should be kept in view.

## Data Availability

Not applicable.

## Appendix

Fathers’ Questionnaire

1. Was this the first time you attended child birth? y/n
2. What was your Motivation?
  - Own wish
  - Partners wish
  - Other reasons
3. Did you know what to expect during child birth? y/n
4. Did your expectation come true? Y/n
5. Did you feel involved? y/n
6. Did you have the impression that your presence was beneficial?
  - No, for nobody
  - Yes for my partner
  - Yes for me personally
  - Yes for my child
  - Yes for our relationship
7. Did you have the impression, that your partner needs you? y/n
8. Did you have the impression, that you were able to support your partner? y/n
9. Did you feel helpless?
10. Were you overwhelmed
11. Did you feel fear?
12. Did you feel happiness having attended child birth? y/n
13. Did you feel traumatized? y/n
14. Would you attend another child birth?
15. What would have been nice to have during labor?
16. Do you have any advice for a becoming father?
